# Lost (Poetry)

**Published:** 1876-07

**Authors:** Rose Terry Cooke


### *Lost.*

BY ROSE TERRY COOKE.

Once on a time she came to me,
As some small star from heaven might flee—
To be a mortal's sole delight,
A love by day, a dream by night.
The sweetest thing on land or sea,
My little darling crept to me.

A trembling, tender, fairy thing,
Too grave to smile, too sad to sing.
Aware of earth with grieved surprise,
An alien from her native skies,
A baby angel strange to see,
My little darling came to me.

But love and loving taught her smiles,
And life and living baby wiles—
The way to cling, to coax, to kiss,
To fill my soul with deepest bliss ;
My heart of hearts, my life was she,
This little love who came to me.

What words she stammered, soft and low
No other ear but mine could know ;
More gentle than a cooing dove,
More fond than any voice of love,
So shy, so sweet, so tenderly
My little darling spoke to me.

I know not how to tell the grace
That dwelt upon her wistful face—
The tinted skin, the lip's pure bloom,
The clearest eyes that knew not gloom,
The hair as soft as mote wings be,
My little darling showed to me.

Alas ! I know that all is gone,
That here I sit and grieve alone,
That every fair and gracious thing
I loved and lost is but a sting ;
Another thorn thy memory,
My little darling brings to me.

But kindly night doth pity pain ;
In all my dreams she comes again ;
Her precious head is on my breast ;
My happy arms caress her rest ;
I hear her words of tender glee ;
My little darling kisses me.

Ah ! sweet is night—too sweet, too brief—
When day recalls our bitterest grief,
The hungry heart, the longing dire,
That burns the soul with vain desire,
The ancient cry of wild distress,
The Rachel-mourning, comfortless,
O God ! once more that face to see !
My little darling come to me !

—*Harper's Magazine for April.*

